# Biothermodynamic Assay of *Coptis-Evodia* Herb Couples

**DOI:** 10.1155/2015/565364

**Published:** 2015-08-30

**Authors:** Hongbo Yang, Min Su, Qi Yao, Yanling Zhao, Danhong Chen, Lei Jia

**Affiliations:** ^1^Yunnan Institute of Materia Medica, Kunming 650111, China; ^2^Institute of Chinese Materia Medica, PLA 302 Hospital, Beijing 100039, China

## Abstract

*Objective.* To illustrate the difference in cold/hot natural properties and therapeutic effect of *coptis-evodia* herb couples by using cold/hot plate differentiating technology and microcalorimetry combined with material basis analysis *in vivo* and *in vitro*. It showed that animal retention ratio in hot pad significantly decreased along with the decrease in *coptis* proportion in *coptis-evodia* herb couples. In addition, Zuojin wan markedly reduced the retention ratio of gastritis mice in the hot pad, while Fanzuojin wan displayed an opposite result. Further, Mg^2+^-ATPase, Ca^2+^-ATPase, and T-AOC activity significantly weakened in *coptis*-treated group in the livers of the mice. In the gastric cells from the gastritis mice, Fanzuojin wan remarkably increased calorific value for growth and metabolism, while Zuojin wan significantly reduced the calorigenic effect. It suggested that the changes in the major chemical compositions (especially alkaloids) were the material base-induced transformation between “cold” and “hot” syndromes. The material basis which affected the transformation between “cold” and “hot” syndromes might be X_2_, X_3_, X_4_, X_8_, epiberberine hydrochloride, jatrorrhizine hydrochloride, coptisine sulphate, palmatine hydrochloride, and berberine hydrochloride. The CHPD combined with microcalorimetry technology is a good method to determine the differences in the “cold” and “hot” natural properties of *coptis-evodia* herb couples.

## 1. Introduction

The “cold” (Han) or “hot” (Re) property of traditional Chinese medicine is determined by its therapeutic effect on “cold” or “hot” syndrome which involves physiological, biochemical, metabolic, and pathological changes [1, 2].

A study has shown that “cold” medicines significantly suppress thyroid, adrenal, ovaries, and other endocrine systems, while “hot” drugs enhance the functions of these endocrine systems in animal experiments [3]. A kind of “cold” drug containing anemarrhena and gypsum was successfully used to copy a “cold” rat model, and a “hot” drug containing aconite, ginger,* Codonopsis*, and* Astragalus* cured the “cold” symptom. Some studies suggested that luteinizing hormone, thyroid-stimulating hormone, and adrenocorticotropic hormone levels significantly elevated in the “cold” animal model, while the “hot” drugs attenuated the increased hormone levels [4–6].

Clinical studies have shown low basal metabolism in patients with “cold” syndrome and high metabolism in the “hot” patients. It was proposed that that “cold” or “hot” syndrome might be a typical reaction of the body and the “cold”/“hot” drugs could change the current state [7]. Since mitochondria are major organelles providing cells with energy, succinic dehydrogenase (SDH) activity increases in the status of being “hot,” suggesting a positive correlation between “hot” symptom and body energy metabolism. As expected, after being treated with the “cold” drugs, the SDH activity was significantly attenuated, facilitating the recovery of mitochondrial respiration in liver. It has been well documented that SDH, adenosine triphosphatase (ATPase), and adenosine kinase (ADK) activity is significantly enhanced in “hot” rats compared with the “cold” ones.

*Coptis-evodia* herb couples including Zuojin wan, Ganlu san, Zhuyu wan, and Fanzuojin wan are composed according to different proportions. It is well known that* Coptis-evodia* herb couples mainly are used to treat gastrointestinal diseases. Gastric acid secretion inhibition, affecting gastrointestinal motility, and analgesic and anti-inflammatory properties have been widely reported in Zuojin wan and its similar formulae. Further, c-fos and corticotropin releasing hormone (CRH) mRNAs markedly are downregulated after Zuojin wan treatment. And Zuojin wan could elevate the gastric PH value and ulcer index (UI) [8, 9]. It was confirmed that Zuojin wan or Ganlu san efficiently eliminated “hot” or aggregated “cold” symptom. Furthermore, Zuojin wan and Ganlu san significantly attenuated Na^+^-K^+^-ATPase and Ca^2+^-Mg^2+^-ATPase activity in rat's erythrocyte membrane and reduced serum interleukin-6 (IL-6) and stimulating hormone (TSH) levels.

In the present study, we used “cold”/“hot” plate differentiating (CHPD) technology combined with biothermodynamics analysis to determine the transformation of “hot” and “cold” syndromes* in vivo* and* in vitro* in the absence or in the presence of* coptis-evodia* herb couples. Further, the components for the activity materials were also analyzed by ultra performance liquid chromatography (UPLC) fingerprints [10–12]. Based on this, we expect to build an efficient method to determine the “hot” and “cold” properties of traditional Chinese medicines* coptis-evodia* herb couples.

## 2. Materials and Methods

### 2.1. Preparation of* Coptis-Evodia* Herb Couples

*Rhizoma Coptidis* and* Fructus Evodiae* used in the present study were purchased from Beijing Lvye Pharmaceutical Co., Ltd. (batch number 20070601). They were identified to be dried rhizomes of* Coptis chinensis Franch*. and ripe fruit of* Evodia rutaecarpa (Juss.) Benth* by Professor Xiong Xiaohe, Research Institute of TCM, PLA 302 Hospital (Beijing, China).

The herbs were washed, dried, and crushed to powder: weighed* Rhizoma Coptidis* and* Fructus Evodiae powder*, respectively. Then weighted different proportions (*Rhizoma Coptidis* and* Fructus Evodiae*) of Zuojin wan similar formulae are prepared according to the following criteria: 10 : 1 and 6 : 1 for Zuojin wan, 4 : 1 and 2 : 1 for Ganlu san, 1 : 1 for Zhuyu wan, 1 : 2, 1 : 4, and 1 : 6 for Fanzuojin wan. The weight of each sample was 210 g. The samples were added in 10 volumes of deionized water at 40°C for 30 min and extracted for three times (2 h for 10 times amount of water, 1 h for 8 times amount of water, and 0.5 h for 6 times amount of water). After that, the extracts were combined and concentrated under reduced pressure at 75°C. Finally, the extracts were dried under vacuum drying conditions at 50°C until constant weight.

### 2.2. Main Reagents

ATPase, total antioxidant capability (T-AOC), and superoxide dismutase (SOD) biochemical assay kits were purchased from Nanjing Jiancheng Bioengineering Institute (Nanjing, China).

### 2.3. Experimental Animals

Specific pathogen-free (SPF) KM mice weighing 18–22 g were provided by the Experimental Animal Center of Academy of Military Medical Sciences (Beijing, China). The animal room was maintained at 22 ± 2°C and 30%–60% relative humidity. The rats were given free access to food and water. All the experiments were conducted in accordance with the national guidelines for the care and use of laboratory animals. This study was approved by the Ethnic Committee of Affiliated Hospital of Kunming University of Science and Technology (Kunming, China).

### 2.4. Temperature Tropism Assay in Normal Mice

**Figure 1:**
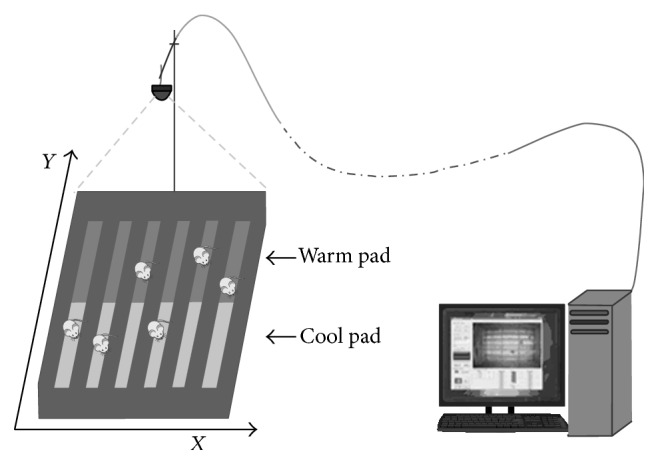
Intelligent animal temperature tropism behavior monitoring system.

The normal mice were divided into a vehicle group, a* coptis* group (5.0 g/kg), a Zuojin wan group (5.0 g/kg), a Ganlu san group (5.0 g/kg), a Zhuyu group (5.0 g/kg), a Fanzuojin wan group (5.0 g/kg), and a Zhuyu wan group (5.0 g/kg) (6 animals in each group). The animals were administrated for 7 days and once a day. 30 min after the administration, the animals were placed in a temperature tropism intelligent monitoring instrument (patent number ZL2008200004444.2) (Figure 1) [13–16]. At room temperature of 20 ± 2°C, the cold or hot pad was set at 25°C or 40°C, respectively. When the actual temperature is reached, 6 mice (labeled with 1→6) were placed within different channels of the instrument. The mice were tracked by the software camera (15 frames per second). The experiment was repeated for 7 days and once a day. Retention ratio is residence time in hot pad/the total monitoring time *∗* 100%.

### 2.5. Temperature Tropism Assay in Cold Sodium Hydroxide-Induced Gastric “Cold” Model and 10% Pepper-Ethanol Solution-Induced Gastric “Hot” Model

The mice were divided into a vehicle group, a “cold” model group, a “cold” model plus Zuojin wan group (5.0 g/kg), a “cold” model plus Ganlu san group (5.0 g/kg), a “cold” model plus Zhuyu wan (5.0 g/kg) group, a “cold” model plus Fanzuojin wan (5.0 g/kg) group, a “hot” model group, a “hot” model plus Zuojin wan group (5.0 g/kg), a “hot” model plus Ganlu san group (5.0 g/kg), a “hot” model plus Zhuyu wan (5.0 g/kg) group, and a “hot” model plus Fanzuojin wan (5.0 g/kg) group.

To establish gastric “cold” symptom, the animals were given cold water (4°C) for three days and once a day. After fasting for 24 h, the mice were administrated with 4°C NaOH (0.3 mol/L, 10 mL/kg). Then they received various treatments for 7 days and once a day. To copy gastric “hot” symptom, the animals were administrated with 10% ethanol pepper solution (20 mL/kg) for 3 days and once a day. 10% pepper-ethanol solution was prepared as follows. Pepper oil was purchased from Tashuifang Co., Ltd. 3 mL of pepper oil was then dissolved by 10% ethanol solution to reach a total volume of 30 mL. After that, the animals received various treatments for 7 days and once a day.

### 2.6. General Status of Animals

During the experiment, the body, food intake, water intake, and oxygen consumption of the mice were recorded for 7 days successively.

### 2.7. Biochemical Assay of Live Tissue

Prepared 10% liver homogenates and performed biochemical assay of Na^+^-K^+^, Mg^2+^, Ca^2+^ATPase, T-AOC, and SOD are in accordance with the instructions of the manufacturers by using ultraviolet spectrophotometer.

### 2.8. Microcalorimetry Assay of Mouse Gastric Cells [17]

The mice model with gastric “cold” or “hot” symptom was established as described previously. Briefly, the mice were sacrificed by cervical dislocation. Then the peritoneal cavity was open and the stomach was removed and placed in cold normal saline. The stomach was cut off along with the greater curvature. Food debris and blood were washed from the stomach. After that, the cells passing a sheet of cell sieve were cultured in prepared Dulbecco's modified eagle's medium (DMEM).

Under aseptic conditions, DMEM containing gastric cells (appropriate gastric cells from 2 mice weighing 18–22 g) were added to each ampule and then Zuojin wan and its similar formulae were added, respectively. Then the ampule was sealed and placed in a microcalorimetry instrument at a constant temperature of 37°C. The thermogram was not recorded until the curve returns to baseline. Maximum power output (*P*_*m*_) is calculated in accordance with the following formula: *P*_*t*_ = *P*_0_ *e* ^*k*(*t*−*t*_0_)^ (*k* presents cell growth rate constant during the exponential growth phase and *t*_0_ presents the initiation time).

### 2.9. Ultra Performance Liquid Chromatogram (UPLC) Assay of the Material Basis in* Coptis-Evodia* Herb Couples [12]

ACQUITY UPLC BEH C18 column (50 mm × 2.1 mm, 1.7 *μ*m) (Waters, Milford, USA) was used for this part experiment. The column temperature was set at 22 ± 0.5°C. The mobile phase was 0.05% phosphoric acid/water (v/v)-acetonitrile. The measurement wave was 270 nm. The injection volume was 1 *μ*L. Number of theoretical plates was more than 3000 according to berberine hydrochloride.

## 3. Results

### 3.1. Zuojin Wan Increases the Retention Ratio of Normal Mice in Hot Pad

**Figure 2:**
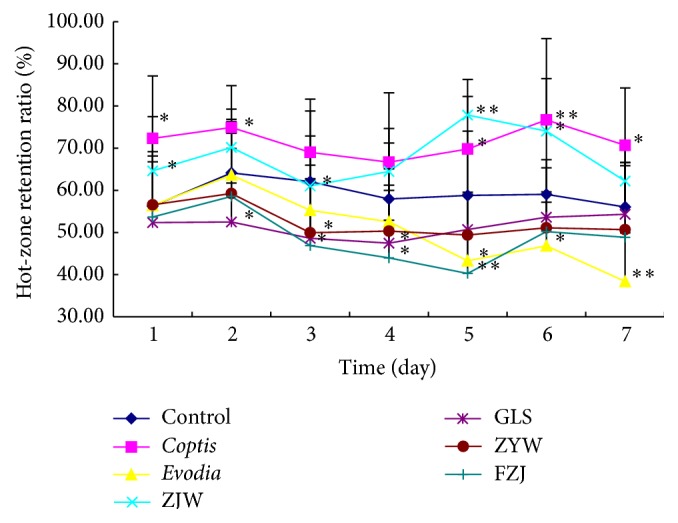
Retention ratio of normal mice in hot pad after the treatments of* coptis-evodia* herb couples. Data are presented as mean (*n* = 6) ± SD. ZJW: Zuojin wan; GLS: Ganlu san; ZYW: Zhuyu wan; FZJ: Fanzuojin wan. ^*∗*^ *P* < 0.05, ^*∗∗*^ *P* < 0.01 versus control (*t*-test).

It is shown that the retention ratio in the hot pad decreased along with the decrease in* coptis* proportion in* coptis-evodia* herb couples. The order of the retention ratio in the hot pad was* coptis* > Zuojin wan > Ganlu san ≈ Zhuyu wan > Fanzuojin wan >* evodia*. The results demonstrated that Zuojin wan had a significant thermotaxis effect, while Fanzuojin wan and* evodia* markedly increased the tendency to being cold in normal mice (Figure 2).

### 3.2. General Status of Normal Mice after Being Treated with* Coptis-Evodia* Herb Couples

**Figure 3:**
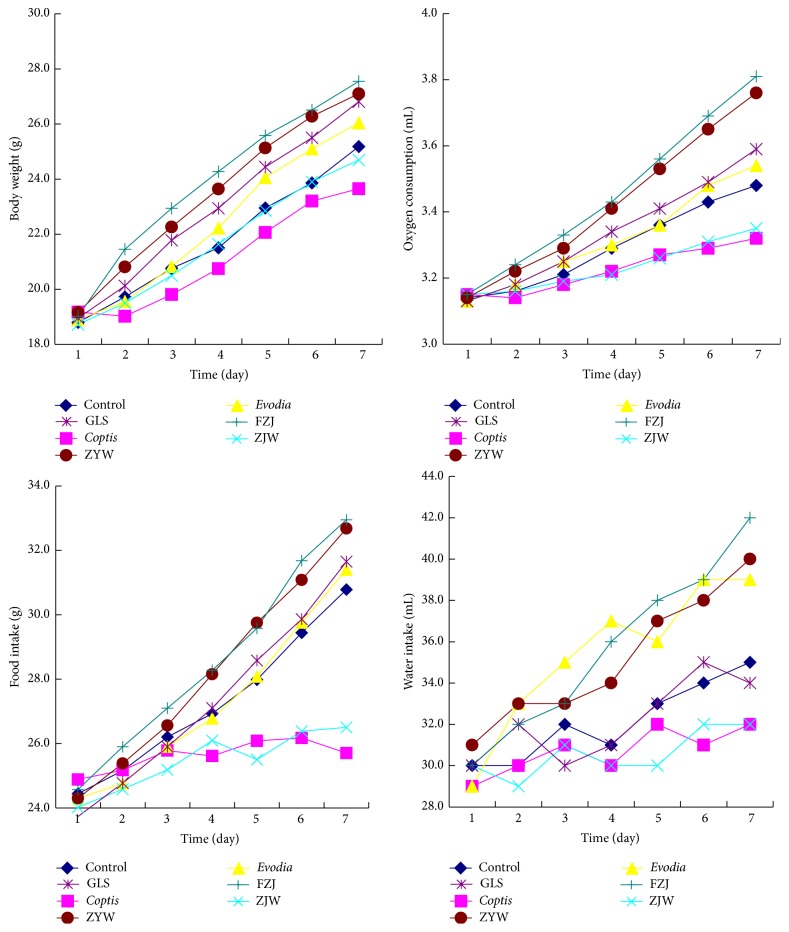
General status of normal mice after the treatments of* coptis-evodia* herb couples. ZJW: Zuojin wan; GLS: Ganlu san; ZYW: Zhuyu wan; FZJ: Fanzuojin wan.

The body weight, food intake, and water intake significantly increased in the normal mice treated with Zhuyu wan and Fanzuojin wan, while the oxygen consumption markedly reduced in Zuojin wan-treated mice (Figure 3). Furthermore, the difference was enlarged along with the prolongation of the time. In addition, Ganlu san and* evodia* had no similar effects.

### 3.3. Changes in Related Biochemical Parameters in the Liver of Normal Mice Treated with* Coptis-Evodia* Herb Couples

The Mg^2+^-ATPase, Ca^2+^-ATPase, and T-AOC activity was significantly weakened in* coptis*-treated group, while being enhanced in* Evodia*-, Fanzuojin wan-, and Zhuyu wan-treated groups. Compared with the control, Fanzuojin wan significantly attenuated the SOD activity in the liver, while Zhuyu wan remarkably enhanced the SOD activity (Table 1).

### 3.4. The Establishment of Mice Model with Gastric “Cold” or “Hot” Symptom

**Figure 4:**
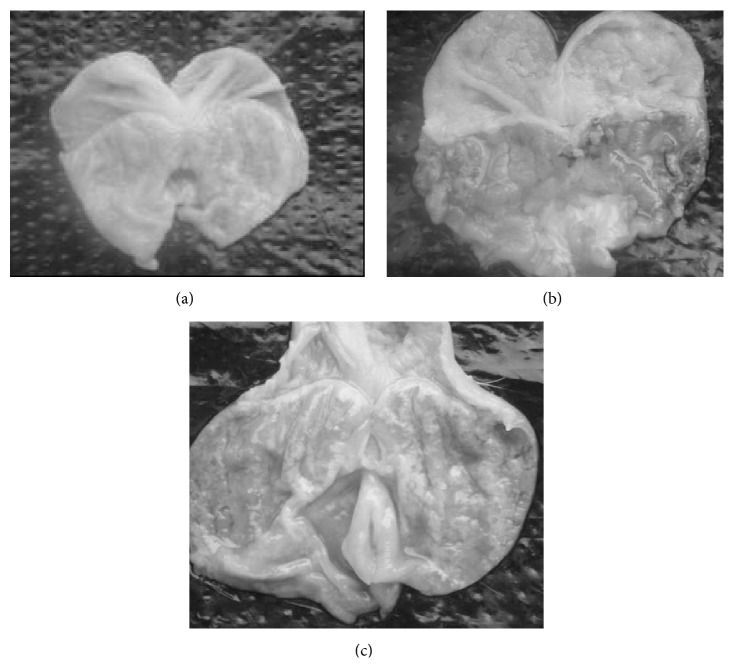
Naked eye observation of stomachs of mice. (a) Normal mouse; (b) mouse with gastric “cold” symptom; (c) mouse with gastric “hot” symptom.

The normal mouse stomach was smooth and pink. However, red and white gastric mucosa, red or purple surface damage, and clear vascular permeability were observed in the mice with gastric “cold” symptom. Meanwhile, significant congestion and ulcers were seen in the mice with “hot” symptom (Figure 4).

### 3.5. Zuojin Wan Increases Retention Ratio of Mice with Gastric “Hot” Symptom, While Fanzuojin Wan Increases Retention Ratio of Mice with Gastric “Cold” Symptom in Hot Pad

**Figure 5:**
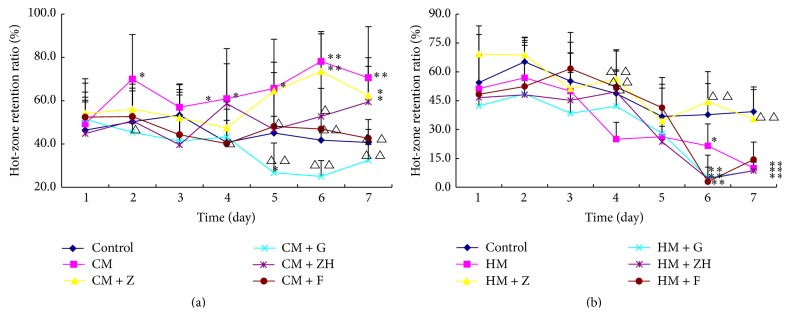
Retention ratio of mice in hot pad after treatments of* coptis-evodia* herb couples. Data are presented as mean (*n* = 6) ± SD. (a) CM: cold model; Z: Zuojin wan; G: Ganlu san; ZH: Zhuyu wan; F: Fanzuojin wan; (b) HM: hot model; Z: Zuojin wan; G: Ganlu san; ZH: Zhuyu wan; F: Fanzuojin wan. ^*∗*^ *P* < 0.05, ^*∗∗*^ *P* < 0.01 versus control; ^△^ *P* < 0.05, ^△△^ *P* < 0.01 versus CM or HM (ANOVA).

The results revealed that the retention ratio in hot pad was significantly higher in the mice with gastric “cold” symptom than that in the normal controls. In the mice with gastric “cold” symptom, Ganlu san, Zhuyu wan, and Fanzuojin wan significantly reduced the retention ratio in hot pad at 4 day (Figure 5(a)). In the gastric “hot” model, Zuojin wan markedly increased the animal retention ratio in hot pad compared with the model group. And the effect was gradually enhanced along with the prolongation of the time (Figure 5(b)).

### 3.6. General Status of Gastric “Cold” or “Hot” Mice after Being Treated with* Coptis-Evodia* Herb Couples

**Figure 6:**
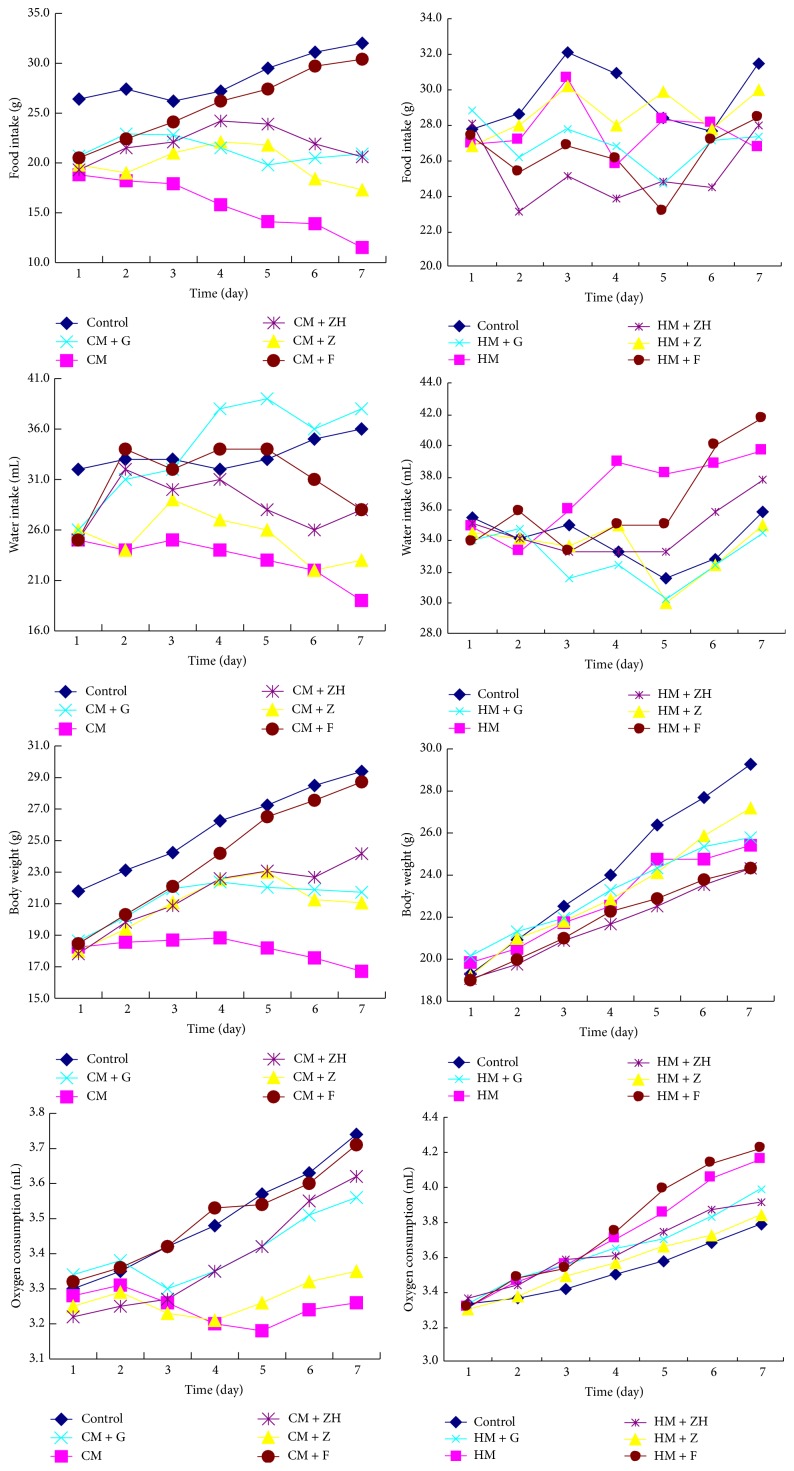
General status of mice in hot pad after treatments of* coptis-evodia* herb couples. CM: “cold” model; HM: “hot” model; Z: Zuojin wan; G: Ganlu san; ZH: Zhuyu wan; F: Fanzuojin wan.

The water intake significantly increased and the water intake remarkably decreased in mice with gastric “hot” symptom. The body weight was slowly increased in both animal models (Figure 6).

Zuojin wan significantly increased food intake and body weight and decreased water intake in the mice with gastric “hot” symptom, while Fanzuojin wan markedly increased food intake, body weight, and water intake in the mice with gastric “cold” symptom (Figure 6).

The oxygen consumption was markedly reduced in Zuojin wan-treated gastric “cold” mice, while being increased in Fanzuojin wan-treated animals (Figure 6).

### 3.7. Changes in Related Biochemical Parameters in the Liver of Gastric “Cold” or “Hot” Mice Treated with* Coptis-Evodia* Herb Couples

The Na^+^-K^+^-, Mg^2+^-, Ca^2+^-ATPase, T-AOC, and SOD activity was significantly weakened in the gastric “cold” mice but enhanced in the gastric “hot” mice (Table 2).

Fanzuojin wan markedly enhanced the Na^+^-K^+^-, Mg^2+^-, and Ca^2+^-ATPase activity in the gastric “cold” mice, while Fanzuojin wan attenuated the activity of these enzymes. Ganlu san, Zhuyu wan, and Fanzuojin wan significantly weakened Na^+^-K^+^ and Mg^2+^-ATPase activity in the gastric “hot” mice (Table 4).

Fanzuojin wan and Ganlu san significantly reduced the T-AOC and SOD activity of the mice with gastric “hot” symptom. And Zhuyu wan and Fanzuojin wan markedly enhanced the T-AOC and SOD activity of the gastric “cold” mice (Table 2).

### 3.8. Thermogenic Assay of Normal Mouse Gastric Cells Treated with* Coptis-Evodia* Herb Couples

**Figure 7:**
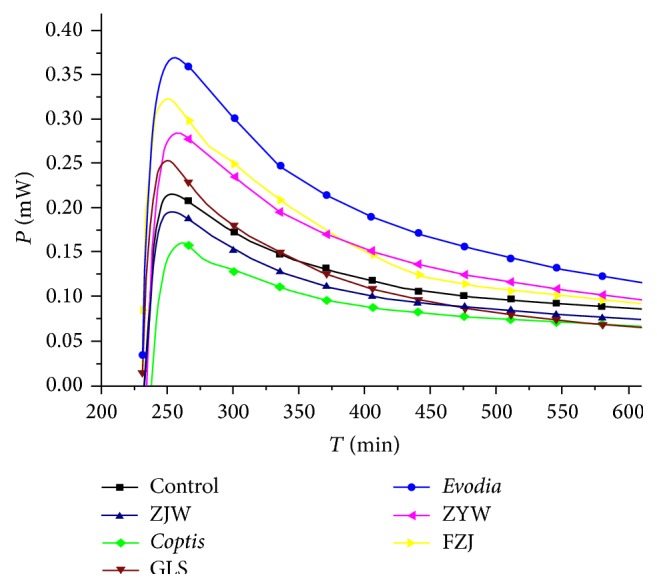
Thermogenic curves of normal mouse gastric cells treated with* coptis-evodia* herb couples. ZJW: Zuojin wan; GLS: Ganlu san; ZYW: Zhuyu wan; FZJ: Fanzuojin wan.

The thermogenic curve was shown in Figure 7 and maximum power output (*P*_*m*_) data were present in Table 3. It was found that the *P*_*m*_ was gradually increased with the increase in evodia proportion. The *P*_*m*_ in FZJ-treated normal mouse gastric cell was higher than that in ZJW-treated cell.

### 3.9. Thermogenic Assay of “Cold” or “Hot” Symptom Mouse Gastric Cells Treated with* Coptis-Evodia* Herb Couples

**Figure 8:**
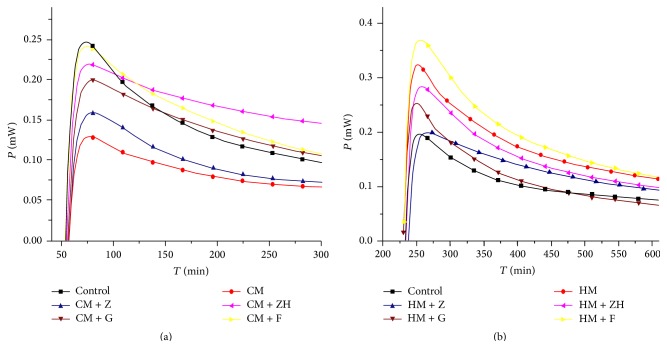
Thermogenic curves of gastric “cold” or “hot” mouse gastric cells treated with* coptis-evodia* herb couples. CM: “cold” model; HM: “hot” model; Z: Zuojin wan; G: Ganlu san; ZH: Zhuyu wan; F: Fanzuojin wan.

The thermogenic curve was shown in Figure 8 and maximum power output (*P*_*m*_) data were present in Table 4. It revealed that Fanzuojin wan increased *P*_*m*_ in “cold” symptom mouse gastric cells, whereas Zuojin wan decreased *P*_*m*_ in “hot” symptom mouse gastric cells.

### 3.10. UPLC Assay of the Material Basis in* Coptis-Evodia* Herb Couples

**Figure 9:**
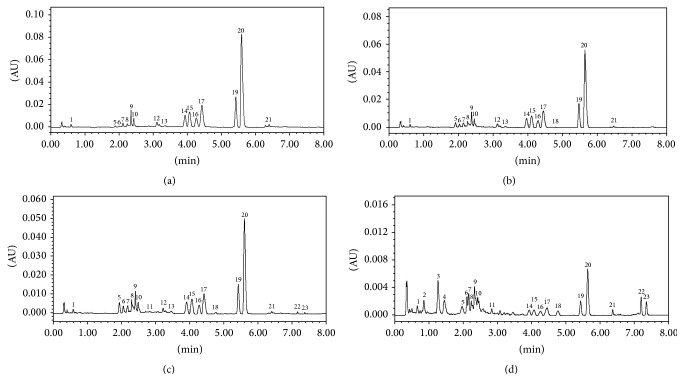
Fingerprint of Zuojin wan and its formulae. (a) Zuojin wan; (b) Ganlu san; (c) Zhuyu wan; (d) Fanzuojin wan. 11—hydroevodiamine; 15—epiberberine; 16—jatrorrhizine; 17—coptisine; 19—palmatine; 20—berberine; 22—evodiamine; 23—rutaecarpine; other unknown materials.

According to the information provided by the HPLC fingerprints of* coptis-evodia* herb couples, we compared the fingerprints and confirmed 23 specific fingerprint peaks. Among them, the area of 14 peaks was about 90% of the total area. Thus, these 14 peaks were confirmed to be specific peaks (Figure 9).

**Figure 10:**
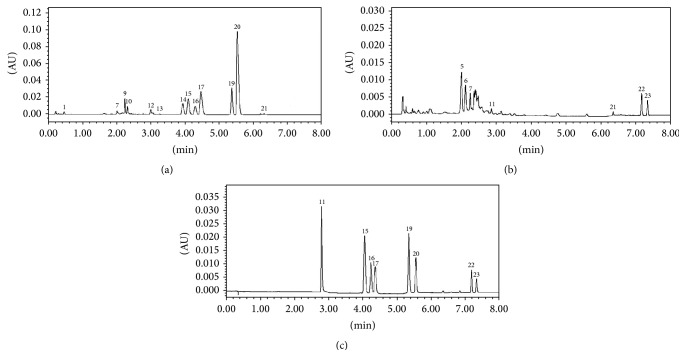
Chromatogram of* coptis*,* evodia*, and mixed standard solution. (a):* coptis*; (b):* evodia*; (c): mixed standard solution. 11—hydroevodiamine; 15—epiberberine; 16—jatrorrhizine; 17—coptisine; 19—palmatine; 20—berberine; 22—evodiamine; 23—rutaecarpine; 1–10, 12–14, 18, and 21—other unknown materials.

For the area of berberine hydrochloride peak (wave 20#) was largest in the total area (10% or more) with the highest peak height and relative stability, it was selected for the control wave. Under the above chromatographic conditions, 1 *μ*L of* coptis-evodia* herb couples solutions was injected to record the chromatograms (Figure 10).

After that, we used external standard method to calculate alkaloids dissolution rate per unit area of a single herb in berberine, evodia, and Zuojin wan and its similar formulae (Table 5).

Among four similar formulae, the resemblance of Zuojin wan, Ganlu san, Zhuyu wan, and Fanzuojin wan to control fingerprint was gradually decreased, suggesting differences in chemical compositions among the four formulae.

## 4. Discussion

To the best of our knowledge, it is first time for us to investigate the “cold” and “hot” properties of* coptis-evodia* herb couples by biothermodynamics method. Biothermodynamics is a science focusing on energy transfer and thermal variations in metabolic process of life systems [18, 19]. Its principal idea is displaying the energy metabolism process by the means of thermodynamic functions [19].

Energy metabolism in a living body system will change in the presence of traditional Chinese medicine (TCM). Thus, it is necessary to obtain the changes in thermodynamic parameters to reflect the differences in biological activity of various reagents measured.

Microcalorimetry (MCM) is an important method for biothermodynamics. Currently, it is becoming a key approach to studying body metabolism characteristics and rules, preliminary activity screening of drugs, drug interactions, and taxonomic identification [20–23]. In the present study, we used MCM in the cold/hot properties of TCM in the early studies [24].

In this study, we selected* coptis-evodia* herb couples for the biothermodynamic assay.* Coptis-evodia* herb couples are consisted of* berberine* and* evodia*. The therapeutic effect is completely different if the proportion changes. Secondly, the active ingredients (mainly alkaloids) are identified. Thirdly, the two herbs* berberine* and* evodia* have distinct “cold” and “hot” properties. Thus, this formula is typical and representative in some degree.

In the temperature tropism experiment, we found that animal retention ratio in hot pad decreased along with the decrease in coptis proportion in* coptis-evodia* herb couples. In the mice with gastric “cold” symptom, Ganlu san, Zhuyu wan, and Fanzuojin wan significantly reduced the retention ratio in hot pad, while in the gastric “hot” model Zuojin wan markedly increased the animal retention ratio in hot pad compared with the model group. Meanwhile, related biochemical parameters changed in the normal, “cold” symptom, and “hot” symptom mice. These above results reflected differences in “cold” and “hot” properties of Zuojin wan and its similar formulae.

*In vitro* study revealed thermogenic changes in* coptis-evodia* herb couples. It showed that* coptis-evodia* herb couples had different effects on the growth and metabolism of the gastric cells. Zuojin wan reduced the heat production in the gastric cells from the mice with gastric “hot” symptom, while Fanzuojin wan increased the heat production in the gastric “cold” symptom cells. From the principal component analysis, we could draw that the difference in the formulae was determined by thermodynamic parameters like *R* and *P*_*m*_.

Principal component analysis by UPLC showed that the composition differences in* coptis-evodia* herb couples were X_2_, X_3_, X_4_, X_8_, epiberberine hydrochloride, jatrorrhizine hydrochloride, coptisine sulphate, palmatine hydrochloride, and berberine hydrochloride. Further, changes in dissolution rate might be involved in them. These might be the material basis for the transfer of “cold” and “hot” properties in* coptis-evodia* herb couples.

In summary, the CHPD combined with microcalorimetry technology is a good method to determine the differences in the “cold” and “hot” natural properties of* coptis-evodia* herb couples. Further, UPLC is efficient to confirm the material basis.

## Figures and Tables

**Table 1:** Related biochemical parameters in the liver of the normal mice after *coptis-evodia* herb couples treatment ($\overset{-}{x}\pm s$, *n* = 6).

Group	Na^+^-K^+^-ATPase (*μ*mol·mg^−1^·h^−1^)	Mg^2+^-ATPase (*μ*mol·mg^−1^·h^−1^)	Ca^2+^-ATPase (*μ*mol·mg^−1^·h^−1^)	T-AOC (U/mgprot)	SOD (U/mgprot)
Control	5.13 ± 0.40	5.22 ± 0.48	6.11 ± 0.41	0.2351 ± 0.0437	89.38 ± 9.56
*Coptis*	4.92 ± 0. 36	3.78 ± 0.60^*∗∗*^	4.75 ± 0.44^*∗∗*^	0.1696 ± 0.0523^*∗*^	86.31 ± 13.22
ZJW	4.84 ± 0.34	4.59 ± 0.23	5.10 ± 0.35^*∗*^	0.2003 ± 0.0340	99.21 ± 11.27^*∗*^
GLS	5.10 ± 0.52	4.77 ± 0.49	5.61 ± 0.31	0.2591 ± 0.0580	92.64 ± 11.82
ZYW	5.66 ± 0.36^*∗*^	5.12 ± 0.32^*∗∗*^	6.09 ± 0.60^*∗∗*^	0.3428 ± 0.0845^*∗∗*^	100.15 ± 14.44^*∗*^
FZJ	5.73 ± 0.39^*∗∗*^	6.33 ± 0.63	7.10 ± 0.48^*∗*^	0.3594 ± 0.0595^*∗∗*^	82.67 ± 12.53
*Evodia*	5.44 ± 0.80	5.39 ± 0.58^*∗*^	6.60 ± 0.60^*∗∗*^	0.3608 ± 0.0270^*∗∗*^	89.99 ± 10.44

ZJW: Zuojin wan; GLS: Ganlu san; ZYW: Zhuyu wan; FZJ: Fanzuojin wan. ^*∗*^ *P* < 0.05, ^*∗∗*^ *P* < 0.01 versus control (*t*-test).

**Table 2:** Changes in related biochemical parameters in the liver of the mice with gastric “cold” or “hot” symptom after being treated with *coptis-evodia* herb couples ($\overset{-}{x}\pm s$, *n* = 6).

Group	Na^+^-K^+^-ATPase (*μ*mol·mg^−1^·h^−1^)	Mg^2+^-ATPase (*μ*mol·mg^−1^·h^−1^)	Ca^2+^-ATPase (*μ*mol·mg^−1^·h^−1^)	T-AOC (U/mgprot)	SOD (U/mgprot)
Control	4.38 ± 0.72	3.96 ± 0.48	3.72 ± 0.63	0.2381 ± 0.539	87.36 ± 12.11
CM	2.58 ± 0.44^*∗∗*^	1.97 ± 0.23^*∗∗*^	2.57 ± 0.25^*∗∗*^	0.1515 ± 0.0357^*∗*^	68.37 ± 8.39^*∗∗*^
CM + ZJW	2.43 ± 0.38^*∗∗*^	1.87 ± 0.36^*∗∗*^	2.45 ± 0.49^*∗*^	0.1244 ± 0.0721^*∗∗*^	64.81 ± 15.00^*∗∗*^
CM + GLS	3.13 ± 0.53^*∗*^	2.04 ± 0.25^*∗∗*^	2.29 ± 0.38^*∗∗*^	0.1493 ± 0.0435^*∗*^	72.02 ± 11.13^*∗*^
CM + ZYW	3.04 ± 0.22^*∗*△^	3.00 ± 0.34^*∗*△△^	2.88 ± 0.42^*∗*^	0.2982 ± 0.0386^△^	98.24 ± 9.37^△△^
CM + FZJ	4.73 ± 0.51^△△^	4.35 ± 0.85^△△^	3.56 ± 0.38^△^	0.2523 ± 0.0265^△△^	85.62 ± 11.08^△^

Control	4.96 ± 0.64	3.84 ± 0.51	3.94 ± 0.67	0.2377 ± 0.254	94.75 ± 10.48
HM	5.63 ± 0.77^*∗*^	5.36 ± 0.66^*∗∗*^	4.97 ± 0.46^*∗*^	0.4168 ± 0.1152^*∗∗*^	148.71 ± 13.20^*∗∗*^
HM + ZJW	4.72 ± 0.70^△^	3.38 ± 0.50^*∗*△△^	3.90 ± 0.54^△^	0.2428 ± 0.1198^△△^	99.36 ± 10.51^△△^
HM + GLS	4.38 ± 0.62^△^	4.33 ± 0.39^△^	4.61 ± 0.38^*∗*^	0.2404 ± 0.0368^△△^	89.28 ± 9.34^△△^
HM + ZYW	5.74 ± 0.82^*∗*^	4.23 ± 0.78^△^	4.53 ± 0.17^*∗*^	0.5644 ± 0.0269^*∗∗*△^	122.58 ± 10.98^*∗*△^
HM + FZJ	5.18 ± 0.56^△^	4.64 ± 0.82^*∗*△^	5.09 ± 0.72^*∗*^	0.5676 ± 0.0596^*∗∗*△△^	151.81 ± 17.72^*∗∗*^

CM: cold model; HM: hot model; ZJW: Zuojin wan; GLS: Ganlu san; ZYW: Zhuyu wan; FZJ: Fanzuojin wan. ^*∗*^ *P* < 0.05, ^*∗∗*^ *P* < 0.01 versus control, ^△^ *P* < 0.05, and ^△△^ *P* < 0.01 versus CM or HM (ANOVA).

**Table 3:** The *P*_*m*_ values of normal mouse gastric cell treated with *coptis-evodia* herb couples.

Drug	*P*_*m*_/(mW)
Control	0.216
*Coptis*	0.159
ZJW	0.196
GLS	0.254
ZYW	0.284
FZJ	0.324
*Evodia*	0.370

ZJW: Zuojin wan; GLS: Ganlu san; ZYW: Zhuyu wan; FZJ: Fanzuojin wan.

**Table 4:** The *P*_*m*_ values of “cold” or “hot” symptom mouse gastric cells treated with *coptis-evodia* herb couples.

Drug	*P*_*m*_/(mW) (cold/hot)
Control	0.247/0.196
*Coptis*	0.129/0.324
ZJW	0.159/0.200
GLS	0.200/0.254
ZYW	0.220/0.284
FZJ	0.242/0.370

ZJW: Zuojin wan; GLS: Ganlu san; ZYW: Zhuyu wan; FZJ: Fanzuojin wan.

**Table 5:** Contents of investigated compounds in *coptis*, *evodia,* and *coptis-evodia *herb couples (mg/g).

Alkaloids	*Coptis*	*Coptis-evodia* herb couples				*Evodia*
		Fanzuojin wan	Ganlu san	Zhuyu wan	Fanzuojin wan	
EPI	5.58	3.71	3.00	2.31	0.34	—
JAT	3.45	2.68	2.05	1.57	0.20	—
COP	10.38	6.67	5.03	3.83	0.53	—
PAL	13.54	13.21	8.12	6.01	1.00	—
BER	44.78	36.22	25.61	18.65	2.40	—
HYD	—^a^	+^b^	+	±^c^	0.15	0.17
EVO	—	+	+	0.58	1.78	2.08
RUT	—	+	+	±	0.97	1.23
Total	77.73	62.49	43.81	32.95	7.37	3.48

a: no detection; b: below the detection limit; c: below the quantification limit.
